# Antibacterial activity of Zn-loaded Cuban zeolite against *Helicobacter pylori* in comparison to its Na-loaded and unmodified counterparts

**DOI:** 10.1007/s10653-020-00781-2

**Published:** 2020-11-26

**Authors:** Guido Cerri, Mauro Farina, Antonio Brundu, Elisabetta Gavini, Andrea Salis, Wilfried Dathe

**Affiliations:** 1 Department of Architecture, Design and Urban Planning - GeoMaterials Lab, Sassari University, Via Piandanna 4, 07100 Sassari, Italy; 2 Department of Chemistry and Pharmacy, Sassari University, Via Muroni 23, 07100 Sassari, Italy; 3 Heck Bio-Pharma GmbH, Gerberstraße 15, 73650 Winterbach, Germany

**Keywords:** *Helicobacter pylori*, Clinoptilolite, Mordenite, Zinc, Ammonium, Cation exchange

## Abstract

*Helicobacter pylori* can be found in the stomach of about half of the humans, and a large population can be associated with serious diseases. To survive in the stomach *H. pylori* increases the pH locally by producing ammonia which binds to H^+^ becoming ammonium. This work investigated the effects on the *in-vitro* growth of *H. pylori* of a natural cation-exchanger mainly composed (≈70%) of clinoptilolite and mordenite. The zeolitized material from Cuba was evaluated in its original form (*M*), as well as in its Na- (*M-Na*) and Zn-exchanged (*M-Zn*) counterparts. In the preliminary agar cup diffusion test, *H. pylori* revealed susceptibility only to *M-Zn*, with a direct relationship between concentration and width of inhibition halo. Further experiments evidenced that bacterium replication increases when ammonium is supplied to the growth medium and decreases when zeolites subtract NH_4_^+^ via ion exchange. Due to the multi-cationic population of its zeolites *M* was not effective enough in removing ammonium and, in the Minimum Inhibitory Concentration (MIC) test, allowed bacterial growth even at a concentration of 50 mg/mL. Inhibition was achieved with *M-Na* because it contained sodium zeolites capable of maximizing NH_4_^+^ subtraction, although the MIC was high (30 mg/mL). *M-Zn* evidenced a more effective inhibitory capacity, with a MIC of 4 mg/mL. Zinc has antimicrobial properties and *H. pylori* growth was affected by Zn^2+^ released from clinoptilolite and mordenite. These zeolites, being more selective towards NH_4_^+^ than Zn^2+^, can also subtract ammonium to the bacterium, thus enhancing the efficacy of *M-Zn*.

**Electronic supplementary material:**

The online version of this article (10.1007/s10653-020-00781-2) contains supplementary material, which is available to authorized users.

## Introduction

The gram-negative bacterium *Helicobacter pylori* is very well adapted for colonization of the human stomach, can be found in about half of the human population and seems to belong to the normal human microbiota (Cover and Blaser 2009). This microorganism learned in the evolution of mankind to manipulate the host immune system to its own advantage through unique biochemical mechanisms to persist chronically (Zhang et al. 2020). Due to the interactions between host and cohabitant an overwhelming level of this bacterium increases the risk of chronic gastritis, peptic ulcer and gastric cancer (Abadi and Yamaoka 2018). On the other hand, several studies report that especially in young people *H. pylori* infection reduces allergen-specific immune responses like asthma and other allergic diseases, as well as atopy risk (Hojsak and Kolaček 2014; Taye et al. 2017; Fouda et al. 2018; Zhang et al. 2020). Furthermore, the prevalence of an autoimmune disease like multiple sclerosis (MS) is significantly lower in patients infected with *H. pylori* than in uninfected persons, suggesting that this bacterium acts as a protective factor against the development of MS (Jaruvongvanich et al. 2016). The advantages and the disadvantages of *H. pylori* for humans obviously depend on the level of population of this bacterium.

Due to the dilemma on the correct management of this possibly useful but dangerous bacterium, therapeutic strategies are focused on its eradication. However, *H. pylori* strains resistant to antibiotics such as metronidazole, clarithromycin and levofloxacin already exist (Mosites et al. 2018; Morilla et al. 2019), hence susceptibility tests are recommended before any antimicrobial prescriptions (Abadi and Yamaoka 2018). To increase the eradication rate and reduce the adverse effects of therapy, complementary medicine treatments such as probiotic supplements are also investigated (Eslami et al. 2019; Peng et al. 2019). Another possible way to fight *H. pylori* is to remove the ammonium ions that surround the bacterium. In fact, to colonize the stomach *H. pylori* produces a large amount of urease (Mégraud and Lehours 2007), and this enzyme transforms the urea that diffuses from the blood to the gastric mucosa into CO_2_ and NH_3_; the latter is protonated to NH_4_^+^, which leads to a local rise in pH thus allowing the organism to survive in the acidic gastric environment (Graham and Miftahussurur 2018). Recently, it has been reported that *in-vitro* growth of *H. pylori* can be inhibited by Na-clinoptilolite, which is capable of removing ammonium through the ion exchange Na^+^ ↔ NH_4_^+^ (Farina et al. 2019). Clinoptilolite is the most common of natural zeolites. These aluminosilicates, which are widespread and low-cost minerals, have cation exchange capacity, moreover many zeolites are extremely selective towards ammonium (Pabalan and Bertetti 2001). The properties of natural zeolites can be exploited against *H. pylori* not only because of the possibility of removing ammonium, but also taking advantage of the features of the released cation, which could be chosen among metals with antimicrobial activity.

Based on the above considerations, the present work investigates the effects on *H. pylori* growth of a zeolite-bearing Cuban material, which has been evaluated in its natural form, as well as in its Na- and Zn-exchanged counterparts. The material used for this research is mainly composed of two zeolites, clinoptilolite and mordenite, is known for its high adsorption capacity for histamine (Selvam et al. 2014, 2018), and is already used as medical device in various oral and topical applications (Langbein et al. 2019; Torres et al. 2019; Dathe 2020).

## Experimental

### Materials

The material used here comes from the zeolite deposit located in San Andrés, province Holguín, Cuba, and contains two zeolites, clinoptilolite and mordenite. The supplier employed a conventional mechanical grinding process to prepare a powder with a particle size ~ 40 μm without performing further treatments (Selvam et al. 2014). This zeolite-based material is named '*M*' and aliquots of it were transformed by cation-exchange into the sodium and zinc form, respectively (*M-Na* and *M-Zn*).

*M-Na* was obtained by mixing *M* with a solution of 1 M NaCl (VWR, Ph. Eur. grade, purity 99.9%) setting a solid to liquid ratio of 30 g/L, performing 10 exchange cycles, each of 2 h, at 65 °C under continuous stirring (500 rpm—IKA RCT basic magnetic stirrer equipped with ETS-D5 thermometer). After each cycle, the material was recovered by centrifugation (Hettich Universal 320) and the ion exchange cycle started newly with the precipitant of the former cycle. After the 10th ion exchange cycle, the precipitate was repeatedly rinsed with deionized water up to the complete elimination of the chloride ions (test performed on elutes with AgNO_3_).

*M-Zn* was prepared using *M-Na*, following the same ion-exchange procedure as described above, but substituting the NaCl solution by a 0.5 M solution of ZnSO_4_·7H_2_O (VWR, Ph. Eur. grade, purity > 99%).

### Characterization

The mineralogical composition of the starting material *M* was determined employing a Bruker D5000 diffractometer equipped with a Cu tube, a curved graphite monochromator on the secondary beam, and a point detector. The instrument was set up as follows: 40 kV, 30 mA, 2θ range 2–80°, step size 0.020°, time per step 15 s. The sample was micronized (McCrone mill) after mixing with 20 wt.% of corundum (*α*-Al_2_O_3_, Buehler micropolish II), added as internal standard. The quantities of the mineral phases were determined through the Rietveld method using the software Bruker Topas 5 and taking advantage of the known amount of internal standard. To compare *M*, *M-Na*, and *M-Zn*, the samples were micronized using a Retsch MM400 mill, and their X-ray patterns were collected using a Bruker D2-Phaser diffractometer under the following conditions: 30 kV, 10 mA, CuKα radiation, LynxEye PSD detector with an angular opening of 5°, 2θ range 5.8–70°, step size 0.020°, time per step 2 s, spinner 15 rpm. The diffractograms were evaluated using the software Bruker EVA 14.2 coupled with the database PDF-2 (ICDD).

Chemical analyses of *M*, *M-Na*, and *M-Zn* were performed at the Activation Laboratories Ltd (Actlabs—Ancaster, ON, Canada). Major elements were determined after lithium metaborate/tetraborate fusion of the sample through Inductive Coupled Mass Atomic Emission Spectrometry (ICP-AES), carried out with a Varian Vista 735 ICP; zinc content was measured via ICP Mass Spectrometry (ICP-MS; Perkin Elmer Sciex ELAN 9000) after sodium peroxide fusion. Loss on Ignition (LoI) of the materials were determined through thermogravimetric (TG) analysis with a TA-Instrument Q600 at CeSAR (Centro Servizi di Ateneo per la Ricerca—Sassari University), by heating the samples to 1000 °C (10 °C/min; alumina crucibles; air flow 100 mL/min).

#### *In-vitro* tests

Before experiments, *M*, *M-Na*, and *M-Zn* were sterilized by heating (3 h at 160 °C), then rehydrated for 24 h in a desiccator containing a saturated solution of calcium nitrate (22 ± 1 °C, 53 ± 2% of relative humidity – EBRO EBI-TH1 data logger). Sterilization by heating was effective in obtaining sterilized zeolite (Cerri et al. 2016), and was already used to perform tests with clinoptilolite and *H. pylori* (Farina et al. 2019). The reference strain *Helicobacter pylori* ATCC® 43504™ was used to perform all *in-vitro* tests. The recommendations of Megraud and Lehours (2007) were followed for the *H. pylori* culture. The susceptibility of the bacterium against the zeolite materials was preliminary evaluated by employing the agar cup diffusion method (Saengmee-anupharb et al. 2013; Lawal et al. 2014).

A set of Petri dishes (Ø 80 mm) was prepared by pouring inside each plate 20 mL of Mueller Hinton Agar (Oxoid) supplemented with 5% of defibrinated horse blood (Oxoid). A bacterial suspension with a turbidity equivalent to a 4.0 McFarland standard was prepared using sterile saline (NaCl 9 g/L) and streaked on the solidified culture medium using a sterile swab. Two of the prepared dishes were separated, to be used as control (without zeolite); in the other plates, three cups (Ø 7.9 mm) were excavated inside the agar using the wider end of a pipette tip as an auger. Suspensions (50 µL) of *M*, *M-Na*, and *M-Zn*, prepared with bi-distilled water, were poured into the cups; the materials were tested in triplicate at concentrations of 25, 50 and 100 mg/mL. After about 20 min, the liquid inside the cups was absorbed, and the plates were incubated, upside down, at 37 °C for three days in microaerophilic conditions (85% N_2_, 5% O_2_, and 10% CO_2_—gas mixture obtained with CampyGen™ sachets, Oxoid). After incubation time, the plates were visually checked for bacterial growth. The growth inhibition of *H. pylori* was characterized by the size of the halo around the cup where no bacterial growth occurred. This inhibition zone was measured with a caliper.

In addition to the aforementioned tests only *M-Zn* was subjected to further evaluations with the agar cup method, using the same concentrations of 25, 50 and 100 mg/mL prepared with: i) bi-distilled water, performing 3 replicates for each concentration; ii) Dulbecco’s Phosphate-Buffered Saline without Ca and Mg (pH 7–7.4; Sigma-Aldrich), performing 4 replicates per concentration.

In order to determine the Minimum Inhibitory Concentration (MIC) of the materials, the zeolite powders were dispersed in liquid agar medium (20 mL per plate). For *M* and *M-Na*, the final concentrations amounted to 10, 20, 30, and 50 mg/mL and for *M-Zn* to 0.5, 1, 2, 4 and 6 mg/mL. After the liquid agar medium reached the solid state, two spots of 3 μL each of the bacterial suspension (see above) were applied, and the plates were incubated for 3 days as described. All tests were accomplished in duplicates, and in each experiment a pair of plates without zeolite was used as control. The MIC of zinc ions was determined by the same procedure using ZnSO_4_·7H_2_O (VWR, Ph. Eur. grade, purity > 99%) in final concentrations of 0.14, 0.27, 0.55, 1.10, and 1.6 mg/mL; the zinc ions of these concentrations correspond to the amounts of Zn^2+^ contained in *M-Zn* at concentrations of 0.5, 1, 2, 4 and 6 mg/mL, respectively.

A set of experiments was performed to evaluate the effect of ammonium on the growth of *H. pylori*. Tests were carried out in duplicate using Petri dishes (Ø 54 mm) containing 10 mL of the culture medium. The bacterial growth was evaluated qualitatively by comparing pairs of plates in the presence or absence of 2.3 mg/mL of NH_4_Cl (Sigma-Aldrich; purity 99.5%). The salt was added to the growth medium only in one dish per plate pair getting the following versions:

(i) Petri dishes with and without NH_4_Cl in the culture medium;
(ii) Petri dishes containing *M-Na* with and without NH_4_Cl in the culture medium (*M-Na* concentrations: 46 and 69 mg/mL);
(iii) Petri dishes containing *M* with and without NH_4_Cl in the culture medium (*M* concentrations: 46 and 69 mg/mL).

Zeolite was added to the culture media as described for MIC determinations. The bacterial suspension (prepared as for the agar cup test) was streaked on the solid agar medium with a swab. Plates were incubated as described.

## Results and discussion

The mineralogical composition of the Cuban zeolite *M* is reported in Table 1. Clinoptilolite is the most abundant phase, followed by mordenite, and the two zeolites constitute 68.2 ± 5.5% by weight of the material. The amounts of clinoptilolite and mordenite determined in this sample are similar to those reported earlier by Selvam et al. (2014, 2018) obtained through a semi-quantitative analysis of the same material. Feldspars, chlorite, tridymite, and smectite represent minor components, whereas the amount of amorphous fraction, constituted by volcanic glass, is significant (Table 1); the comparison between observed and calculated X-ray diffraction (XRD) patterns is reported in Electronic Supplementary Material. The presence of smectite traces was described previously in the Cuban zeolite *M* by Cervini-Silva et al. (2016) however, given the small amount, the role of this clay seems to be of minor importance, although is beneficial in children suffering from diarrhea (Madkour et al. 1993).

**Table 1:** Mineralogical composition of *M* in weight percent with estimated standard deviation

Clinoptilolite	Mordenite	Feldspars	Chlorite	Tridymite	Amorphous
40.6 ± 3.2	27.6 ± 2.5	3.2 ± 0.7	2.3 ± 0.5	1.3 ± 0.3	25.0^a^ ± 3.5

^a^It includes traces of smectite (see Supplementary Fig. 1)

**Fig. 1:**
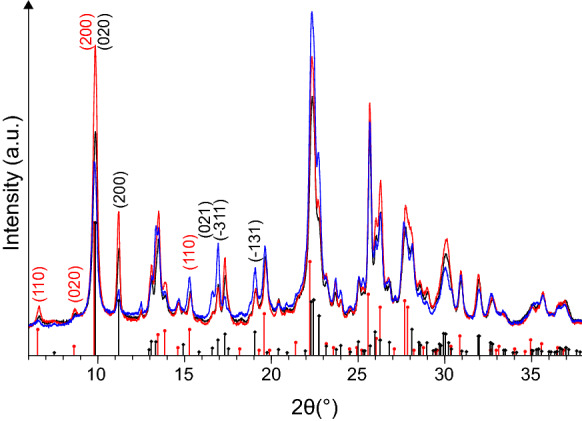
XRD pattern of *M* (black), *M-Na* (red) and *M-Zn* (blue), detail in the 2θ range 6–38°. Black bars: clinoptilolite (PDF N. 80–0464). Red bars: mordenite (PDF N. 29–1257). In brackets, Miller indices of some planes of clinoptilolite (black) and mordenite (red)

The cation exchange process determines some changes in the XRD patterns of clinoptilolite and mordenite (Fig. 1). In particular, in the passage from *M-Na* to *M-Zn* there is a decrease in the intensity of mordenite peaks (110), (020), and (200), along with a slight increase for (310), in agreement with the XRD pattern for sodium mordenite converted into zinc form as reported by Susarrey-Arce et al. (2008). Also in clinoptilolite peaks decrease or increase in intensity passing from the sodium to the zinc form (Fig. 1), and the strongest change concerns the peak (020), which is known to be extremely cation-sensitive (Brundu et al. 2018; Cerri et al. 2018, 2019).

**Fig. 2:**
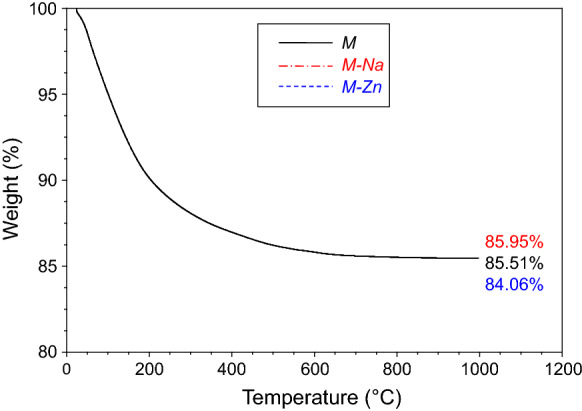
Thermogravimetric analysis (TG) of *M*, *M-Na* and *M-Zn*

Table 2 shows the chemical composition of *M*, as well as that of the same material prepared in the sodium (*M-Na*) and zinc (*M-Zn*) forms. The zinc loaded sample—derived from *M-Na*—evidences low Na, K, Ca and Mg contents, but these "residual quantities" should belong mainly to the amorphous fraction and to the feldspars, which cannot release ions by exchange. This leads to the conclusion that the cation exchange capacity (CEC) of the material was completely exploited in realizing *M-Zn*, which contains 1.92 meq/g of Zn^2+^. The result attained for *M-Na* is slightly lower and amounts 1.85 meq/g of Na^+^. On the other hand, the influence of pretreatment on capacity and selectivity of zeolites for metal ions is known (Semmens and Martin 1988; Pabalan and Bertetti 2001), and a preliminary exchange with sodium proved to be an effective procedure for obtaining homoionic Zn-clinoptilolite (Cerri et al. 2004; Cerri and Brundu 2020). According to Pabalan and Bertetti (2001) and Colella (2005), the CEC of mordenite is comparable to that of clinoptilolite, therefore in *M* (as well as in *M-Na* and *M-Zn*) most exchangeable cations are hosted by clinoptilolite since it is more abundant than mordenite. Smectite can only provide a minimal contribution to the CEC of the material and smectite clays have a lower cation exchange capacity than zeolites (Bish 2013). The difference between *M-Na* and *M-Zn* in terms of LoI (Table 2) is due to the higher water content of the latter one (Fig. 2). In principle, the volume available for H_2_O molecules within zeolite channels corresponds to the portion of microporosity not occupied by cations (Esposito et al. 2015). Thus, the increase in water content from *M-Na* to *M-Zn* makes sense, because each Zn^2+^ replaces 2 Na^+^ and the ionic radius of sodium is larger than that of zinc (Shannon 1976).

**Table 2:** Chemical composition in weight percent of *M*, *M-Na* and *M-Zn*

	SiO_2_	Al_2_O_3_	Fe_2_O_3_	MnO	MgO	CaO	Na_2_O	K_2_O	TiO_2_	P_2_O_5_	ZnO	LoI	Sum
*M*	65.49	11.20	1.60	0.01	1.02	2.61	1.52	1.62	0.24	0.05	–	14.49	99.87
*M-Na*	66.60	11.27	1.13	0.01	0.48	0.18	5.73	0.18	0.24	0.02	–	14.05	99.90
*M-Zn*	63.20	10.43	1.05	< 0.01	0.38	0.19	0.63	0.17	0.21	< 0.01	7.81	15.94	100.00

LoI: Loss on Ignition

**Fig. 3:**
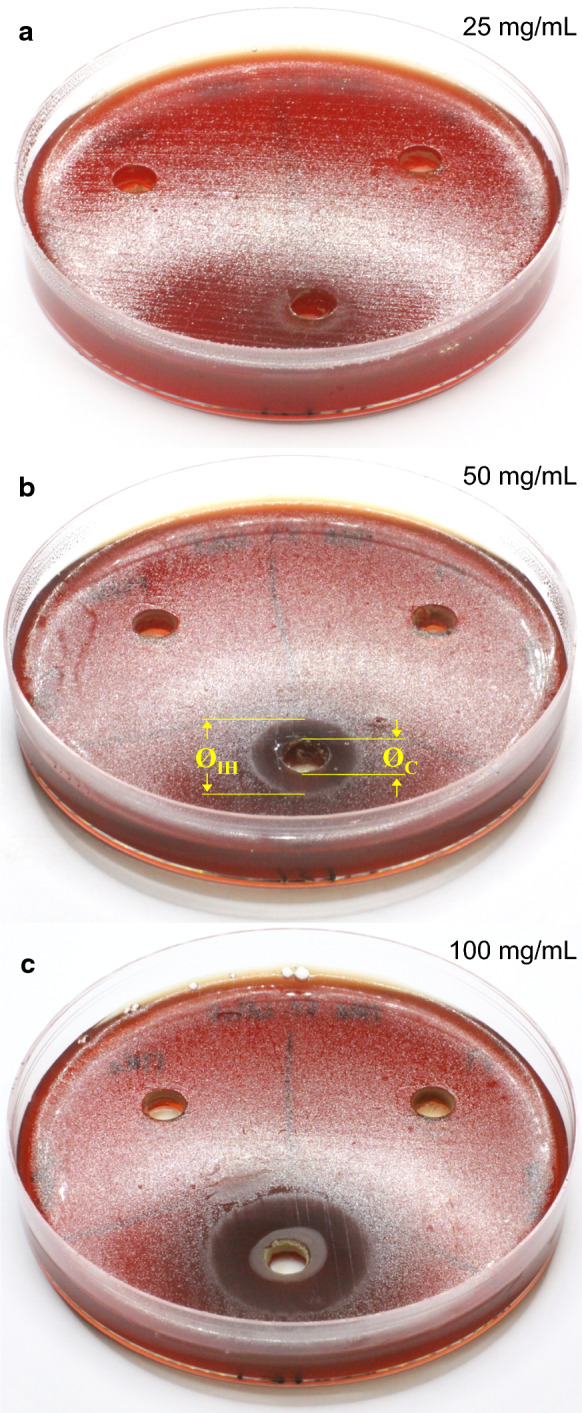
Agar cup test. The zeolite suspensions, having the indicated concentrations, were poured into the cups of each petri dish as follows – top left: *M-Na*; top right: *M*; bottom: *M-Zn*. The inhibition halo was determined as difference between Ø_IH_ and Ø_C_ as shown in **b**

**Fig. 4:**
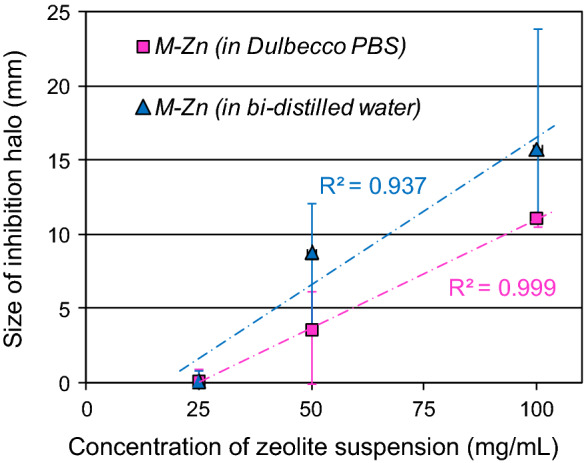
Relationship between size of inhibition halo and concentration of *M-Zn* suspended in bi-distilled water or in Dulbecco Phosphate Buffer Solution

The results of the agar cup test for growth inhibition of *H.* pylori are summarized in Fig. 3. No inhibition halos developed around the cups filled with suspensions of *M* and *M-Na* up to a concentration of 100 mg/mL. In contrast, *H. pylori* resulted sensitive to *M-Zn*, in fact an incipient inhibition of bacterial growth occurred for a concentration of 25 mg/mL (Fig. 3a), and well-defined inhibition halos developed at higher concentrations (Fig. 3b, c). In particular, the size of the halos shows a direct relationship with the concentration of the *M-Zn* suspensions poured into the cups (Table 3). The inhibition zone turned out slightly wider when *M-Zn* was dispersed in bi-distilled water than in Dulbecco phosphate buffer (Fig. 4), albeit halos size showed a greater standard deviation (Table 3). The tiny difference in halos width might depend on the different development of exchange processes during the test. In fact, once suspended in the buffer solution, *M-Zn* can immediately release zinc ions by exchange with Na^+^ and K^+^, therefore the suspension poured into the cups already contained Zn^2+^ in aqueous solution unlike *M-Zn* suspended in bi-distilled water. However, speculations about the small differences in halos size would require further bioassay of the bacterium's susceptibility to the zeolite-bearing materials.

**Table 3:** Growth inhibition of *H. pylori* on the basis of the halo size (mm) around the cups (Ø_IH_—Ø_C_, Fig. 3) containing *M-Zn* suspended in bi-distilled water (6 replicates) or in Dulbecco Phosphate Buffer Solution (4 replicates) at different concentrations

	*M-Zn* conc.: 25 mg/mL			*M-Zn* conc.: 50 mg/mL			*M-Zn* conc.: 100 mg/mL		
	min	max	*average*	min	max	*average*	min	max	*average*
*M-Zn* suspended in bi-distilled water	0.0	0.1	*0.05*	10.2	11.3	*10.8*	15.6	16.1	*15.9*
	0.0	0.0	*0.0*	4.0	5.7	*4.9*	23.7	23.9	*23.8*
	0.0	0.0	*0.0*	10.2	11.2	*10.7*	16.1	16.1	*16.1*
	0.0	0.9	*0.45*	11.8	12.1	*12.0*	13.0	13.2	*13.1*
	0.0	0.0	*0.0*	6.1	7.2	*6.7*	14.8	15.1	*15.0*
	0.0	0.0	*0.0*	7.4	7.9	*7.7*	11.0	11.5	*11.3*
Average values			0.1 (± 0.2)			8.8 (± 2.8)			15.8 (± 4.3)
*M-Zn* suspended in Dulbecco PBS	0.0	0.0	*0.0*	3.4	3.4	*3.4*	11.0	11.5	*11.3*
	0.0	0.0	*0.0*	0.0	0.4	*0.2*	10.9	11.3	*11.1*
	0.0	0.0	*0.0*	5.5	6.2	*5.9*	10.6	11.0	*10.8*
	0.0	1.0	*0.5*	5.0	5.1	*5.1*	11.1	11.6	*11.4*
Average values			0.1 (± 0.3)			3.6 (± 2.5)			11.1 (± 0.2)

Table 4 shows the results related to the determination of the Minimum Inhibitory Concentration (MIC) of the materials. In *M-Zn* a concentration of 2 mg/mL resulted in stunted *H. pylori* growth, whereas inhibition not occurred till 4 mg/mL. With regard to the zinc sulphate solution, stunted bacterial growth was recorded for a concentration of 0.55 mg/mL, and inhibition was achieved at 1.10 mg/mL (Table 4). These results indicate that the antibacterial action is related to zinc. In fact, the MIC of the *M-Zn* suspension is, in terms of zinc concentration, identical to the MIC of the zinc sulphate solution. Thus, the same zinc concentration led to stunted bacterial growth. However, two significant differences should be underlined: (i) in the case of the zeolite suspension, Zn^2+^ became available gradually by cation exchange, whereas in the aqueous solution all Zn^2+^ was accessible from the beginning; (ii) to reach the same concentration of Zn^2+^ present in the zinc sulphate solution, *M-Zn* should release all its zinc content, which is unlikely. In fact, to fully exploit the CEC of the material and obtain *M-Zn*, 10 passages were required in a concentrated solution continuously stirred and kept at 65 °C, whereas the release of Zn^2+^ *in-vitro* took place within a gel during a single step at 37 °C, hence with less favorable conditions for the development of an ion exchange; moreover, also the literature reports the difficulty of obtaining a complete release of zinc (Cerri et al. 2004; Rodríguez-Fuentes 2004; Bonferoni et al. 2007). Despite the two differences mentioned above, *M-Zn* showed the same effectiveness as the zinc sulphate solution.

**Table 4:** Determination of the Minimum Inhibitory Concentration (MIC) of *M*, *M-Na*, *M-Zn* and ZnSO_4_·7H_2_O on the growth of *H. pylori*

	10 mg/mL	20 mg/mL	30 mg/mL	50 mg/mL	
*M*	n	n	n	n	
*M-Na*	n	n	i	i	
	0.5 mg/mL	1.0 mg/mL	2.0 mg/mL	4.0 mg/mL	6.0 mg/mL
*M-Zn*	n	n	s	i	i
	0.14 mg/mL	0.27 mg/mL	0.55 mg/mL	1.10 mg/mL	1.60 mg/mL
ZnSO_4_·7H_2_O	n	n	s	i	i

n—normal growth; s—stunted growth; i—inhibited growth

The antibacterial properties of zinc seem to base on several possible mechanisms of action. An excess of Zn affects life cycle of microbes by extending generation time; moreover, when zinc binds to their membranes, it modulates membrane-associated enzymes, impairs calcium uptake and changes the fluidity of the membrane itself (Goudouri et al. 2014). Zn-exchanged synthetic zeolites acquire antimicrobial properties against a broad range of microorganisms (Demirici et al. 2014), and natural zeolites loaded with zinc exhibit the same ability (Rodríguez-Fuentes 2004). Water disinfection is the main application intended for Zn-exchanged natural zeolites, in particular for clinoptilolite (Top and Ülkü 2004; Hrenovic et al. 2012; Đolić et al. 2017). The antimicrobial effect of Zn^2+^ clinoptilolite against several microorganisms like Gram positive and Gram negative bacteria strains, pathogenic strains like *Vibrio cholera 01* and *Corynebacterium diphtheriae* as well as yeast strains was described by Rodríguez-Fuentes (2004).

The mechanism of Zn^2+^action involves the slow release of the antimicrobial metal ion from the solid phase (Fox et al. 2010). The electrostatic attraction between Zn^2+^ and the negative charge of microbial cell membranes interferes with their permeability and is followed by the penetration of substances into the cell (Ferreira et al. 2016). However, some authors noted antimicrobial action even when the release of Zn^2+^ was very low (Hrenovic et al. 2012; Đolić et al. 2017). They concluded that the metal-loaded zeolites are probably themselves bactericidal, without releasing the ion into the liquid medium (Hrenovic et al. 2012). In this case, a small particle size of the Zn-loaded zeolite is important to intensify contact with microbes and obtain a more efficient disinfectant action (Đolić et al. 2017). However, the antibacterial activity of the latter mechanism was significantly lower than that of free metal cations released in solution from clinoptilolite (Hrenovic et al. 2012).

Previous studies considered the possible use of some zinc compounds to inhibit the growth of *H. pylori*. Chakraborti et al. (2013) demonstrated that zinc oxide nanoparticles functionalized with polyethyleneimine generate ROS (Reactive Oxygen Species) inside bacterial cells with consequent damage to the membrane, degradation of stable RNA, morphological transition to the coccoid form and finally loss of viability. Moreover, zinc ions markedly inhibit the activity of urease, the enzyme produced by *H. pylori* which is crucial for the survival of the bacterium within acidic gastric environment (Pérez-Pérez et al. 1994).

The literature data justify the inhibition of *H. pylori* growth observed in our experiments with zinc solution and *M-Zn*. However, the material containing zeolites in sodium form, *M-Na*, was also able to inhibit the proliferation of *H. pylori*, although it required a higher concentration (30 mg/mL, Table 4). This result coincides with Farina et al. (2019), who employed a clinoptilolite-bearing material whose sodium content was 5.85%, hence comparable to *M-Na* (5.73%). The inhibition determined by Na-clinoptilolite was attributed to its capacity to remove ammonium from the microenvironment of the bacterium via cation exchange, in fact a concentration of 30 mg/mL of NH_4_-clinoptilolite (which could not subtract ammonium because already "saturated") allowed bacterial growth (Farina et al. 2019). Na^+^ ions released by *M-Na* should not inhibit the growth of *H. pylori* because the bacterium is suspended in a physiological solution of NaCl (0.15 M) containing a concentration of Na^+^ three times higher than the MIC of *M-Na*. Table 4 also shows that *M*, the unmodified material, did not inhibit bacterial proliferation, not even at a concentration of 50 mg/mL. In removing ammonium *M* is less effective than *M-Na*, because the latter contains monoionic sodium zeolites and Na^+^ is easily released by clinoptilolite and mordenite, in addition both zeolites exhibit very high preference for NH_4_^+^ over Na^+^ (Pabalan and Bertetti 2001). *M* contains significant amounts of Ca^2+^, Mg^2+^ and K^+^ (Table 2) and experiments conducted on clinoptilolite evidenced that, in ammonium solution, for these cations the exchange process is less effective than for Na^+^ (Rožić et al. 2005). Selvam et al. (2014) came to the same conclusion by testing *M* in buffered and unbuffered solutions.

**Fig. 5:**
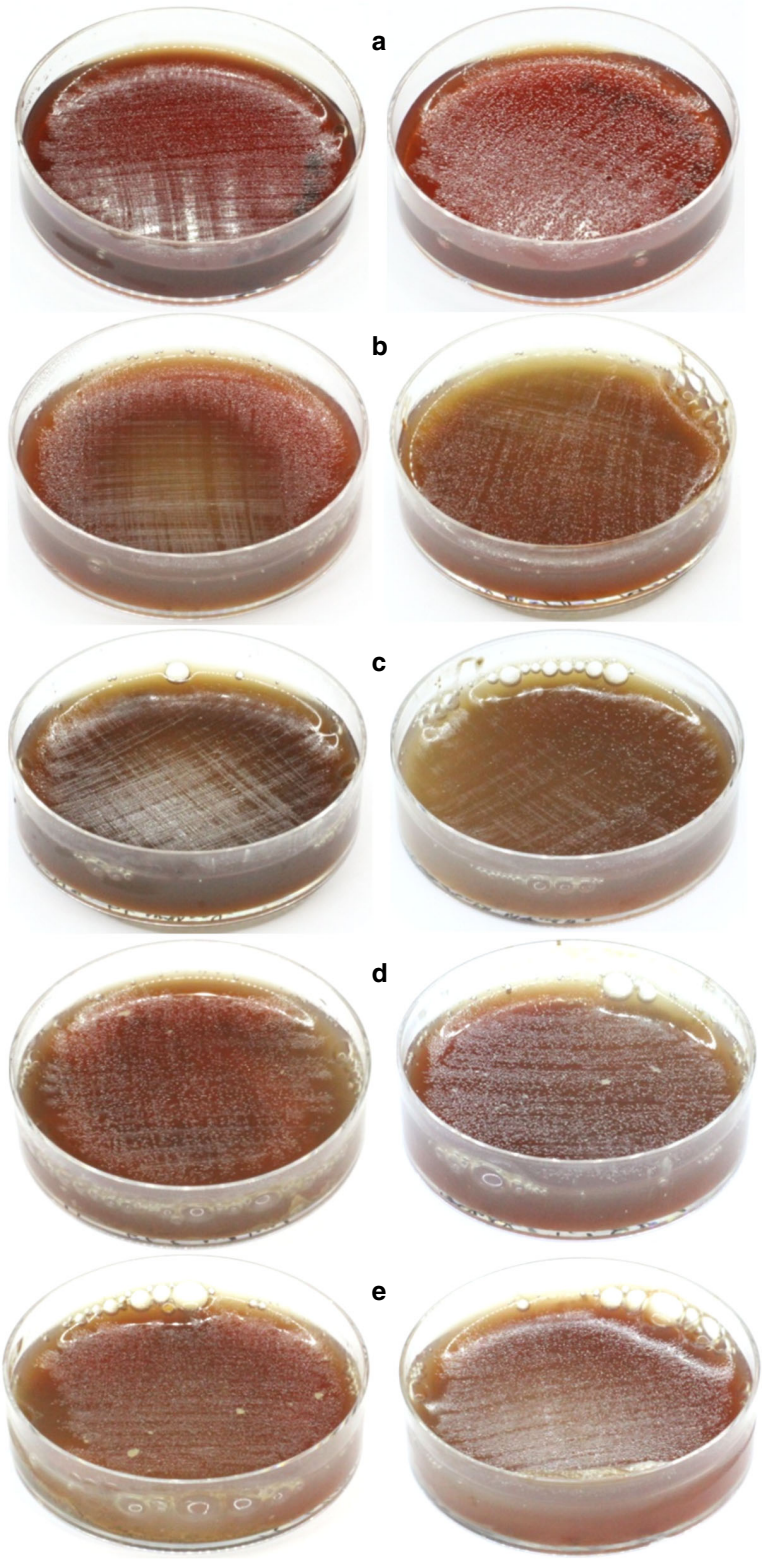
Effect of ammonium salt on *H. pylori* growth: only the plates on the right side contain NH_4_Cl (2.30 mg/mL); **a**—without zeolite; **b**—*M-Na* 46 mg/mL; **c**—*M-Na* 69 mg/mL; **d**—*M* 46 mg/mL; **e**—*M* 69 mg/mL

The relevance of ammonium for *H. pylori* proliferation is shown in Fig. 5. In the zeolite-free plates (Fig. 5a), bacteria grow better in the media containing NH_4_Cl (on the right) than in the control plate (left). It corresponds to Farina et al. (2019), who noted that *H. pylori* colonies in plates containing NH_4_-clinoptilolite seemed to grow better compared with control dishes without this zeolite. Ultimately, the presence of ammonium, supplied through a salt or with a zeolite, seems to promote *H. pylori* proliferation. This is confirmed by the experiments performed with *M-Na*. A concentration of 46 mg/mL affected bacterial growth, which occurred only along the edge of the plate (Fig. 5b, left), whereas in the NH_4_Cl-supplemented media the colonies of *H. pylori* are visible throughout the dish (Fig. 5b, right). An increase of *M-Na* concentration to 69 mg/mL prevented bacterial growth (Fig. 5c, left), unless ammonium was present, a condition that allowed *H. pylori* replication, albeit stunted (Fig. 5c, right). Overall, the progressive reduction in bacterial growth is associated with the increase in the concentration of *M-Na* (Fig. 5a-c, plates on the left side). However, in culture medium without NH_4_^+^ Na-zeolites progressively inhibited *H. pylori* growth, but in ammonium-supplemented media the inhibitory action of *M-Na* was partially reduced, (Fig. 5b-c, plates on the right side). On the contrary, bacterial growth in plates containing *M* at concentrations of 46 and 69 mg/mL (Fig. 5d-e, left) showed no appreciable differences compared to the control plate (Fig. 5a, left). Also in this case, the addition of NH_4_^+^ resulted in an increase of *H. pylori* growth (Fig. 5d-e, right), which appears comparable to that in the control plate integrated with NH_4_Cl (Fig. 5a, right). Thus, *M* is less effective than *M-Na* in removing NH_4_^+^ by exchange, which explains why it was unable to affect bacterial growth. On the other hand, the inhibitory effect determined by the subtraction of ammonium is weak compared to that caused by the release of zinc, in fact the MIC of *M-Na* is 7.5 times higher than *M-Zn* (Table 4). Zn-loaded zeolites can inhibit *H. pylori* growth through two mechanisms: (i) clinoptilolite and mordenite display a marked preference for NH_4_^+^ compared to Zn^2+^ (Blanchard et al. 1984; Barrer and Townsend 1976), resulting in the antimicrobial action of the released zinc and (ii) the inhibitory effect of ammonium removal. This may explain why *M-Zn* showed the same efficacy as the zinc sulphate solution although, probably, not all zinc ions were released from the zeolites.

## Conclusions

The Cuban zeolite-bearing material (*M*) tested in this work is mainly composed (≈70%) of clinoptilolite and mordenite. In its natural form, the material does not inhibit the growth of *H. pylori in-vitro*. On the contrary, the sodium form (*M-Na*) prepared by cation exchange is capable to inhibit bacterial proliferation. The unmodified material (*M*) is not effective enough in removing ammonium to achieve inhibition as, in addition to Na^+^, it contains significant quantities of Ca^2+^, Mg^2+^ and K^+^, cations which are exchanged with greater difficulty.

*In-vitro* experiments evidenced that *H. pylori* replication increases when ammonium is supplied to the growth medium and decreases when NH_4_^+^ is subtracted from the microenvironment of the bacterium. Inhibition can be obtained by ammonium removal using *M-Na*, because clinoptilolite and mordenite in sodium form are capable of maximizing NH_4_^+^ subtraction by ion exchange. However, the MIC of *M-Na* is too high (30 mg/mL) to be of practical use, because achieving this concentration in the stomach, whose volume is about 2 L, would require 60 g of *M-Na*. The material loaded with Zn (*M-Zn*) shows a more powerful inhibitory capacity, with a MIC of 4 mg/mL. Due to its antimicrobial properties, the Zn^2+^ released from the zeolites (clinoptilolite and mordenite) can damage the bacterium cells through different mechanisms. Beside the actions exerted by the released zinc cation, *M-Zn* can also effectively subtract ammonium to the bacterium, in fact both clinoptilolite and mordenite show high preference for NH_4_^+^ over Zn^2+^.

The future direction of this research should be focused on the application of *M-Zn* in clinical trials to determine its efficacy against the proliferation of *Helicobacter pylori*.

## Electronic supplementary material

Below is the link to the electronic supplementary material.

Supplementary file1 (DOCX 91 kb)
