# Supplementary material for: Antibacterial activity of Zn-loaded Cuban zeolite against Helicobacter pylori in comparison to its Na-loaded and unmodified counterparts
Source: Environ Geochem Health. 2020 Nov 26;43(5):2037–48. doi: 10.1007/s10653-020-00781-2 (PMC8081705; doi:10.1007/s10653-020-00781-2)

Antibacterial activity of Zn-loaded Cuban zeolite against *Helicobacter pylori* in comparison to its Na-loaded and unmodified counterparts

Environmental Geochemistry and Health

Guido Cerri*, Mauro Farina, Antonio Brundu, Elisabetta Gavini, Andrea Salis, Wilfried Dathe

*Department of Architecture, Design and Urban Planning - GeoMaterials Lab, Sassari University, Via Piandanna 4, 07100, Sassari, Italy

Corresponding author e-mail: gcerri@uniss.it

Supplementary Fig. 1 – Screenshot of the refinement (software Bruker Topas 5 – Rietveld method) showing the comparison between the observed (blue) and the calculated (red) X-ray powder diffraction pattern for *M*; the grey curve is the difference between the observed and the calculated intensity at each step. To take into account the presence of smectite traces, a "peak phase" at 2θ = 5.69° was inserted, and the quantity of this minor component was joined with that of the amorphous. Corundum (19.96%) was added as internal standard, and the correct amounts of the mineral phases are reported in the Table 1 of the article. Rwp (reliability factor of Rietveld refinement) = 9.546.

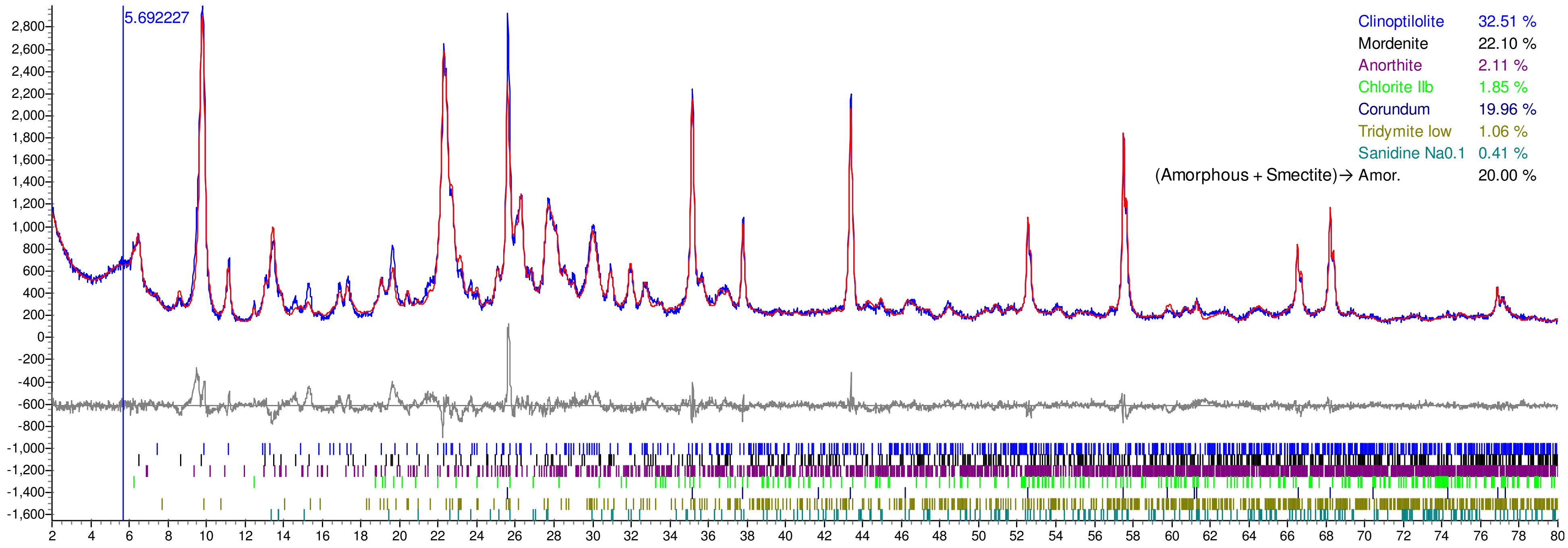

Supplement: Supplementary file 1 — Supplementary file1 (DOCX 91 kb) [file 10653_2020_781_MOESM1_ESM.pdf]
